# Whole genome SNP analysis suggests unique virulence factor differences of the Beijing and Manila families of *Mycobacterium tuberculosis* found in Hawaii

**DOI:** 10.1371/journal.pone.0201146

**Published:** 2018-07-23

**Authors:** Kent Koster, Angela Largen, Jeffrey T. Foster, Kevin P. Drees, Lishi Qian, Edward P. Desmond, Xuehua Wan, Shaobin Hou, James T. Douglas

**Affiliations:** 1 Department of Microbiology, University of Hawaii at Manoa, Honolulu, Hawaii, United States of America; 2 Hawaii State Department of Health, Honolulu, Hawaii, United States of America; 3 Pathogen and Microbiome Institute, Northern Arizona University, Flagstaff, Arizona, United States of America; 4 Department of Molecular, Cellular, and Biomedical Sciences, University of New Hampshire, Durham, New Hampshire, United States of America; 5 California Department of Public Health, Richmond, California, United States of America; 6 Advanced Studies in Genomics, Proteomics and Bioinformatics, Honolulu, Hawaii, United States of America; Institut National de la Recherche Agronomique, FRANCE

## Abstract

While tuberculosis (TB) remains a global disease, the WHO estimates that 62% of the incident TB cases in 2016 occurred in the WHO South-East Asia and Western Pacific regions. TB in the Pacific is composed predominantly of two genetic families of *Mycobacterium tuberculosis* (*Mtb*): Beijing and Manila. The Manila family is historically under-studied relative to the families that comprise the majority of TB in Europe and North America (e.g. lineage 4), and it remains unclear why this lineage has persisted in Filipino populations despite the predominance of more globally successful *Mtb* lineages in most of the world. The Beijing family is of particular interest as it is increasingly associated with drug resistance throughout the world. Both of these lineages are important to the State of Hawaii, where they comprise over two-thirds of TB cases. Here, we performed whole genome sequencing on 82 Beijing family, Manila family, and outgroup clinical *Mtb* isolates from Hawaii to identify lineage-specific SNPs (SNPs found in all isolates from their respective families, and exclusively in those families) in established virulence factor genes. Six non-silent lineage-specific virulence factor SNPs were found in the Beijing family, including mutations in alternative sigma factor s*igG* and polyketide synthases *pks5* and *pks7*. The Manila family displayed more than eleven non-silent lineage-specific and characteristic virulence factor mutations, including in genes coding for MCE-family protein Mce1B, two mutations in fatty-acid-AMP ligase FadD26, and virulence-regulating transcriptional regulator VirS. This study further identified an ancient clade that shared some virulence factor mutations with the Manila family, and investigated the relationship of those and other “Manila-like” spoligotypes to the Manila family with this SNP dataset. This work identified a set of virulence genes that are worth pursuing to determine potential differences in transmission or virulence displayed by these two *Mtb* families.

## Introduction

Despite sustained control efforts, tuberculosis (TB) remains the ninth leading cause of death worldwide [1]. The World Health Organization (WHO) estimated that 62% of the incident TB cases in 2016 occurred in the WHO South-East Asia and Western Pacific Regions [1]. Within those regions, TB in both China and the Philippines in characterized by distinct families of TB–the Beijing family in China and the Manila family in the Philippines [2, 3]. These families fall into global genetic lineages 1 (the Manila family) and 2 (the Beijing family), and are distinct from the lineage 4 clades that are dominant in Europe and North America [4, 5].

The WHO’s Global Tuberculosis Report 2017 indicates these regions and *Mtb* families as areas of concern [1]. The Philippines was only surpassed in number of incident cases by India, Indonesia, and China. As noted in the report, the Fourth National Survey of the Prevalence of TB Disease in the Philippines was conducted from March to December of 2016, and the survey revealed that tuberculosis was not well controlled in the country [1]. Comparing the prevalence of smear-positive TB among participants aged 15 years or more to the case notification rate for smear-positive TB cases in 2016 (142 per 100,000 population) in the same age group gave a prevalence-to-notification ratio of 3.1. From that, the group’s adjusted prevalence of culture positive TB became 463 per 100,000 population (95% CI: 333−592) in 2007 and 512 per 100,000 population (95% CI: 420−603) from that study in 2016. The probability that prevalence did not decline over the period 2007–2016 was estimated at 75%. Following the survey, the estimate of TB incidence in the Philippines was revised to 554 cases per 100,000 (95% CI: 311–866), or 573,000 cases. That was well above the incidence previously estimated by the WHO (322 cases per 100,000). In contrast to the steady or increasing incidence of TB in the Philippines, TB incidence in China is thought to be declining, with the WHO’s estimate being 895,000 cases (64 per 100,000) in 2016.

However, as also noted in the WHO’s report, 4.1% of new TB cases and 19% of re-treated TB cases globally are multidrug-resistant (MDR) TB cases. China is joined by India and the Russian Federation to account for 47% of the world’s total incident MDR TB cases. China saw an estimated 73,000 incident MDR TB cases in 2016, compared to 30,000 for the Philippines. Considerably more concerning is China’s estimated percentage of new TB cases that are MDR in 2016, at 7.1%,compared to 2.6% for the Philippines, though the Philippines’ total MDR rate is expected to continue to increase [6]. Approximately 70% of TB cases in China are of the Beijing family, which also displayed the highest MDR rate of all lineages in the country [7]. Beyond China, it has further been proposed that the population structure of *Mtb* in areas of high drug resistance is shifting towards Beijing family strains [8].

While the Beijing family has been studied with increasing intensity, especially in the context of drug resistance, considerably less work has focused on the Manila family. Both families are of importance to TB control in the United States, where immigration from the WHO South-East Asia and Western Pacific Regions continually presents TB controllers with new TB cases. The State of Hawaii, centrally located in the Pacific and experiencing a high level of immigration from throughout both of those WHO Regions, accordingly has a composition of TB lineages that resembles the United States Affiliated Pacific Islands more than the continental United States [9]. As expected, immigration from Asia imports primarily Beijing family strains to Hawaii, while immigration from the Philippines brings primarily Manila family strains [2, 3]. Those two families combined are responsible for over two-thirds of the TB cases in Hawaii, a ratio that has remained constant since the introduction of the use of spoligotyping to type Hawaii’s *Mtb* in 1997 [10, 11]. Furthermore, high levels of immigration from throughout the Pacific into Hawaii result in the state perennially displaying either the highest or second-highest rates of TB in the United States, with Hawaii’s TB rate remaining steady even as TB rates throughout the US continue to decrease [12].

Thus, the need to further understand the characteristics of the Beijing and Manila families of *Mtb* is of considerable importance not only to countries throughout the Pacific, but also to the United States. Investigating unique differences in virulence factors that are characteristic of each of these families may help explain those families’ success in their originating environments, and may aid in developing improved control strategies if differences affecting latency, reactivation, or host preference are identified. The Manila family, for example, continues to predominate in Filipino hosts, where other, more globally successful lineages have been unable to displace the Manila family and rise to high incidence in the Philippines. Considerably less research has been performed on the Manila family than on the Beijing family or on lineage 4, thus which attributes may contribute to the Manila family’s persistence remains an open question.

This study seeks to utilize next-generation Illumina whole-genome sequencing on a diverse set of Beijing and Manila family-focused clinical *Mtb* isolates from Hawaii in order to identify lineage-specific virulence factor differences of these two families. Here, we examine the *Mtb* genomes to find non-silent (i.e. non-synonymous), single-nucleotide polymorphisms (SNPs) specific to each family in virulence genes as well as other genes of interest. These SNPs are alleles that are unique to either the Beijing or Manila family isolates among the 82 that we studied. In order for a SNP to be described here as a Beijing family-specific SNP, that SNP must have been found in all Beijing family isolates from this study, but not have been found in any of this study’s non-Beijing family isolates. Similarly, SNPs described as Manila family-specific must have been found in all Manila family isolates from this study, but not have been found in any of this study’s non-Manila family isolates.

Additionally, Hawaii and the Pacific region frequently see *Mtb* isolates with spoligotyping fingerprints that resemble the Manila family, but are less commonly encountered than isolates possessing spoligotyping patterns that have been established as “Manila family.” This study will utilize phylogenetics from full-genome sequences to better describe the relationship of such patterns and isolates to the Manila family.

## Materials and methods

### Selection of *Mtb* isolates for study

A comprehensive database of all 1,076 *M*. *tuberculosis* isolates processed for genetic fingerprinting for the State of Hawaii from 2002 through 2016 (and including a partial set from 2017) was obtained from the State of Hawaii Department of Health Tuberculosis Control Program for this study. Spoligotype octal codes are presented as recorded by the Centers for Disease Control and Prevention (CDC) contracted reference laboratory that performed the typing. Lineage or family names were assigned to spoligotype octal codes using the SpolDB4 database, with “EAI2_MANILLA” entries being considered “Manila family.” [13]. Eighty-two *Mtb* isolates from this set were selected for use in this study (Table 1). Additionally, a *Mycobacterium bovis* BCG P3 isolate was used as an outgroup for phylogenetic analysis, as *M*. *bovis* has been shown to have diverged from the *M*. *tuberculosis* ancestor prior to the differentiation of the TB lineages examined in this study [5].

**Table 1:** Full list of sequenced *Mycobacterium tuberculosis* isolates.

#	Accession Number	Spoligotype Name	Spoligotype Octal Code	MIRU-VNTR Loci 1–12	MIRU-VNTR Loci 13–24	Comments
**7**	09L1688	Beijing	000000000003771	223315175533	444444423348	Beijing Diversity Selection
**10**	09L2178	Beijing	000000000003771	222325173533	445644423328	Beijing Clustered
**16**	10L8643	Beijing	000000000003771	222325173533	445644423326	Beijing Clustered
**18**	10L9505	Beijing	000000000003771	223325173432	345544423539	Beijing Diversity Selection
**22**	11L1883	Beijing	000000000003771	222325173533	445644423326	Beijing Clustered
**28**	11L0426	Beijing	000000000003771	222325173533	445644423328	Beijing Clustered
**29**	11L0427	Beijing	000000000003771	222325173533	445644423328	Beijing Clustered
**35**	12L6290	Beijing	000000000003771	222325173533	445644423326	Beijing Clustered
**38**	12L7610	Beijing	000000000003771	223325173533	545534223328	Beijing Diversity Selection
**39**	12L7621	Beijing	000000000003771	223325173521	343544423339	Beijing Diversity Selection
**40**	12L7991	Beijing	000000000003771	222325173533	445644423328	Beijing Clustered
**52**	15RF4481	Beijing	000000000003771	223325163533	448544423328	Beijing Clustered
**56**	15RF6753	Beijing	000000000003771	222325173533	445644423328	Beijing Clustered
**58**	16RF0102	Beijing	000000000003771	222325173533	445644423328	Beijing Clustered
**63**	16RF2356	Beijing	000000000003771	222325173533	445644423328	Beijing Clustered
**64**	16RF4738	Beijing	000000000003771	223325163533	448544423328	Beijing Clustered
**75**	08L7125	Beijing	000000000003771	222325173533	-	Beijing Clustered
**79**	10L8839	Beijing	000000000003771	222325173533	445644423326	Beijing Clustered
**80**	10L9812	Beijing	000000000003771	222325173533	445644423326	Beijing Clustered
**81**	11L3224	Beijing	000000000003771	222325173533	445644423326	Beijing Clustered
**82**	12L6284	Beijing	000000000003771	222325173533	445644423326	Beijing Clustered
**85**	13L2956	Beijing	000000000003771	222325173533	445644423328	Beijing Clustered
**86**	16RF7084	Beijing	000000000003771	222325173533	445644423328	Beijing Clustered
**74**	08L0161	Beijing	000000000003751	222325173533	-	Uncommon Beijing
**77**	09L2141	Beijing	000000000003751	222325173533	445644423328	Uncommon Beijing
**2**	05L3477	Manila	677777477413771	254326223432	14a843263217	Manila Clustered
**3**	05L3487	Manila	677777477413771	254326223432	14a943263217	Manila Clustered
**4**	08L7789	Manila	677777477413771	254326223432	14a923273217	Manila Diversity Selection
**6**	09L1025	Manila	677777477413771	265326223432	148443263216	Manila Diversity Selection
**9**	09L1898	Manila	677777477413771	254326223432	14a843263217	Manila Clustered
**11**	09L2431	Manila	677777477413751	254316223432	14a743263217	Manila Diversity Selection
**14**	10L0115	Manila	677777477413771	254326223432	149843263217	Manila Diversity Selection
**15**	10L5331	Manila	677777477413671	254326223432	147743263217	Manila Diversity Selection
**17**	10L9503	Manila	677777477413771	244326223432	149443263217	Manila Diversity Selection
**20**	11L1563	Manila	677777477413771	254326222432	245843263217	Manila Diversity Selection
**23**	11L3658	Manila	677777477413771	254326223332	148943273217	Manila Diversity Selection
**24**	11L4599	Manila	677777477413771	254326223432	14a843243215	Manila Diversity Selection
**34**	12L6289	Manila	677777477413771	234326223432	176843263217	Manila Diversity Selection
**41**	12L9260	Manila	677777477413771	254326223432	13A843263217	Manila Diversity Selection
**43**	13L1313	Manila	677777477413771	244326223442	14A943263215	Manila Diversity Selection
**45**	13L2435	Manila	677777477413771	254326223432	14A943263217	Manila Clustered
**46**	13L2950	Manila	677777477413771	254326223432	14A843263217	Manila Clustered
**48**	14RF3136	Manila	677777477413771	254326223432	14a943263217	Manila Clustered
**49**	14RF3267	Manila	677777477000771	254326223432	149743263217	Manila Clustered
**50**	14RF5521	Manila	677777477413771	254326223432	14a843253217	Manila Clustered
**51**	15RF0460	Manila	677777477413771	255326223432	147843263217	Manila Clustered
**53**	15RF5637	Manila	677777477413771	254326223432	14a923263217	Manila Clustered
**59**	16RF0108	Manila	677777477413771	255326223432	147843263217	Manila Clustered
**60**	16RF1671	Manila	677777477413771	254326223432	14a943263217	Manila Clustered
**61**	16RF1679	Manila	677777477413771	254326223432	14a943253217	Manila Clustered
**62**	16RF1689	Manila	677777477413700	254326223432	14a943263217	Manila Clustered
**65**	16RF5423	Manila	677777477413771	254326223422	247843263217	Manila Clustered
**69**	09L0735	Manila	677777477413771	-	-	Singleton, Deletions Study
**70**	09L1111	Manila	677777477413771	-	-	Singleton, Deletions Study
**89**	96128	Manila	677777477413701	-	-	Uncommon Manila
**90**	20034	Manila	677777477413751	-	-	Uncommon Manila
**5**	09L0897	EAI5	777777776413771	364225223533	245273243416	For Manila-like Comparison
**33**	11L5411	EAI5	777777776413771	275225223532	245273243415	For Manila-like Comparison
**84**	13L2584	no match*	677774477413771	244326223432	14A843263217	Manila-like Clustered
**21**	11L1565	no match**	600777477413771	254326223432	146743263217	Manila-like Cluster 1
**30**	11L2683	no match**	677777402003771	251326223432	14b843233217	Manila-like Cluster 2
**37**	12L7424	no match**	677777402003771	251326223432	14B843233217	Manila-like Cluster 2
**44**	13L1569	no match**	600777477413771	254326223432	146743263217	Manila-like Cluster 1
**8**	09L1897	no match	777777607360371	123326143326	342224123235	No Match Diversity Selection
**42**	13L0560	no match	703717740003771	2254251b3533	524244223345	No Match Diversity Selection
**71**	04L0177	no match	737777377413771	264225223532	-	No Match Diversity Selection
**72**	06L6898	no match	737777377413771	264225223532	-	No Match Diversity Selection
**73**	06L7696	no match	737777377413771	264225223532	-	No Match Diversity Selection
**13**	09L4214	H3	777777777720771	225325153323	232634423337	Lineage 4 Diversity Selection
**27**	10L8137	H3	777777770020771	222225153322	143334213328	Lineage 4 Diversity Selection
**76**	09L2140	H3	777777770020771	122325143323	233334213326	Lineage 4 Diversity Selection
**78**	10L6616	H3	777777770020771	122325143323	233334213326	Lineage 4 Diversity Selection
**1**	04L0176	LAM9	777777607760771	123326153326	-	Lineage 4 Diversity Selection
**19**	11L0415	T1	777777777760771	242325142222	241544223124	Lineage 4 Diversity Selection
**31**	11L2831	T1	777777777760771	2233151433–3	343544223326	Lineage 4 Diversity Selection
**36**	12L7116	T1	777777777760771	233326153311	232334222523	Lineage 4 Diversity Selection
**12**	09L3451	T3	377737777760771	223326153323	242334223425	Lineage 4 Diversity Selection
**47**	13RF4795	U	777777760000000	223325143322	242324223422	U Diversity Selection
**54**	15RF5640	U	777777760000000	223325143322	242324223422	U Diversity Selection
**55**	15RF6749	U	777777760000000	223325143322	242334223322	U Diversity Selection
**57**	15RF6755	U	777777760000000	223325143322	242324223422	U Diversity Selection
**83**	13L2363	U	777777760000000	223325143322	242324223422	U Diversity Selection
**66**	BCG P3	BOVIS1_BCG	676773777777600	-	-	*M*. *bovis* outgroup
-	Reference	H37Rv	777777477760771	2x3226133321	242534233525	Reference for alignment

Complete list of *Mycobacterium tuberculosis* isolates selected for sequencing for this study. Isolates are identified by one or two digit University of Hawaii DNA extraction numbers and Centers for Disease Control and Prevention (CDC) genome accession numbers. Spoligotypes are named as presented in SpolDB4.

*No match in SpolDB4, but strongly suspected to be Manila family based on similarity.

**Manila-like.

#### Selection of *Mtb* isolates for identification of lineage-specific virulence factor differences

From the database, 25 Beijing family (spoligotype 000000000003771) isolates covering a chronological range from 2005 to 2016 were selected for whole genome sequencing. Of those, 19 isolates were selected from putative transmission clusters, four were selected to increase our represented Beijing family diversity (based on their MIRU-VNTR [Mycobacterial Interspersed Repetitive Unit-Variable Number Tandem Repeat] fingerprints), and two were selected due to possessing an uncommon Beijing family spoligotype (000000000003751). Thirty-one Manila family (representative spoligotyping pattern: 677777477413771) isolates were selected, of which 15 were selected from putative transmission clusters and 12 were selected based on uniqueness of MIRU-VNTR fingerprints to maximize this study’s Manila family diversity. To further increase the diversity of Manila family isolates examined in this study, two additional Manila family isolates with uncommon Manila family spoligotyping patterns (isolates 96128 and 20034) and two Manila family isolates with unique large-sequence-polymorphism (LSP) deletion patterns from a prior study (09L0735 and 09L1111) were selected from our laboratory’s Mycobacterial DNA Bank at the University of Hawaii, where their extracted DNA was archived [14]. Fourteen outgroup isolates from non-Beijing, non-Manila lineages from Hawaii during the 2002–2016 time period were selected for comparison, primarily on the basis of uniqueness of spoligotyping and MIRU-VNTR patterns. These isolates included four with the H3 spoligotype, one LAM9, three T1, one T3, and five from a suspected outbreak cluster with an undefined spoligotyping pattern (777777760000000). An additional three-isolate suspected transmission cluster with no SpolDB4 spoligotype match (737777377413771) was also selected.

#### Selection of *Mtb* isolates for comparison of “Manila-like” strains

Two putative two-isolate transmission clusters whose spoligotypes had no match in SpolDB4, but whose spoligotyping patterns otherwise exhibited substantial similarity to the Manila family, were selected for whole-genome comparison to the Manila family (Manila-like Cluster 1: isolates 21 and 44; Manila-like Cluster 2: isolates 30 and 37). Two EAI5 isolates were also selected (isolates 5 and 33) for sequencing due to the EAI5 clade’s similarity to the Manila family. An additional clustered isolate with no spoligotype match in SpolDB4 (isolate 84) that we considered to be Manila family based on the similarity of its spoligotype and MIRU-VNTR fingerprints was also included. Finally, two isolates with no spoligotype match in SpolDB4 and no near-matches with MIRU-VNTR patterns from other isolates (isolates 8 and 42) were selected to represent lineage-agnostic outgroups.

### Whole genome sequencing

#### Recall of state of Hawaii *Mtb* isolates

All required isolates were requested from the laboratories where they had been previously sent by the State of Hawaii for contracted fingerprinting, and where they were archived (California Department of Public Health State Laboratory and Michigan Department of Community Health). Spoligotyping and 24-loci MIRU-VNTR fingerprints are displayed as provided by those laboratories. The Michigan Department of Community Health returned extracted DNA from their isolates, while isolates from the California Department of Public Health State Laboratory were returned as “double-killed” sample preps using a treatment of immersion in 70% ethanol and then heating at 80°C for one hour.

#### DNA extraction and whole genome sequencing

DNA extraction was performed as previously described by the National Institute of Public Health and Environmental Protection (RIVM), Bilthoven, The Netherlands (Isolation of Genomic DNA from Mycobacteria Protocol), or according to the source state laboratory’s internal protocol. DNA was quantified with the Qubit 2.0 dsDNA Broad Range Assay. Genomic libraries were prepared using the Illumina Nextera XT DNA Library Kit using manual normalization and sequenced on the Illumina MiSeq Platform with v3 Chemistry for 300 bp paired-end reads.

### Data analysis

SNP matrices were produced using a modification of the NASP pipeline, with Bowtie2 used for alignment, GATK used for SNP-calling, and SNPs being filtered for a minimum of ten-fold read coverage and 90% read consensus [15, 16, 17]. Repetitive regions were removed by the NASP pipeline. A *M*. *tuberculosis* type strain H37Rv genome (GenBank NC_000962.3) was used as the scaffold for read alignment for identification of virulence factor SNPs, and our *de novo* sequenced representative Manila family genome “96121” (GenBank CP009427) was used as the scaffold for read alignment for construction of phylogenies and comparison of “Manila-like” strains. The number and percentage of mapped reads was determined using SAMtools flagstat. Percent coverage of the 4,411,532 base pair H37Rv reference genome NC_000962.3 was determined using SAMtools mpileup.

#### Identification of lineage-specific virulence factor differences

Output matrices from the NASP pipeline were imported into a Microsoft Access database and SQL queries were utilized to identify SNPs relative to H37Rv that were shared by all members of either the Beijing or Manila families that we sequenced. SQL queries were further used to search all non-Beijing isolates for those SNPs in order to exclude them as non-specific to the Beijing family if found. For the Manila family, SNPs that were shared by all Manila family isolates were searched for in all non-Manila family isolates–however, their presence in Manila-like isolates or isolates with no spoligotype match in SpolDB4 did not automatically exclude them, as the relationships of those isolates to the Manila family was examined separately and individually. SNPs in genes described in Forrellad et al.’s 2013 review of *Mtb* virulence were designated as “virulence factor SNPs” [18]. The strength of the association between lineage-specific and lineage-characteristic SNPs and their respective groups versus outgroups was calculated using the Fisher Exact method using R Statistics version 3.4.1 [19].

#### Exploring the relationship of Manila-like isolates to the Manila family

Spoligotypes of isolates (presented in this work as their CDC octal codes) were compared in order to identify the number of gained or lost spacers relative to the representative Manila family spoligotype (677777477413771). All isolates examined were individually aligned against Manila family *do novo* sequenced genome 96121 using the NASP pipeline to determine the numbers of SNPs separating those isolates from 96121. Putative Manila family-specific non-silent virulence factor SNPs were examined for all Manila-like, EAI5, and no-match isolates, with *Mtb* type strain H37Rv and the Beijing family serving as known outgroups.

A phylogenetic tree was constructed using MEGA7 utilizing maximum parsimony, obtaining the most parsimonious tree using the Subtree-Pruning-Regrafting algorithm with search level 2 where the initial trees were obtained by the random addition of sequences with ten replicates, and branch lengths were calculated using the average pathway method [20]. The maximum parsimony phylogeny was supported by 1,000 bootstrap repetitions, and rooted with *Mycobacterium bovis* BCG P3. To further support that phylogeny, a model averaged phylogeny was generated using jModelTest 2.1.10 with likelihood scores computed using a maximum likelihood optimized base tree with NNI base tree search and 11 substitution schemes [21]. Akaike Information Criterion (AIC), Akaike Information Criterion corrected for small sample sizes (AICc), and Bayesian Information Criterion (BIC) (all with model averaging and 100% confidence intervals) were evaluated to find the best-fit. The model-averaged phylogeny was created using AICc as the criterion for tree weights and with majority rule consensus and a 100% confidence interval.

Isolates were designated as “ancestral” or “modern” on the basis of the presence or absence of the TbD1 deletion region [22]. The presence of the TbD1 deletion was identified in the sequenced isolates by a gap in the bases aligned to *de novo* sequenced genome 96121 (GenBank CP009427) in the range of nucleotide positions 1,759,200 to 1,761,320.

## Results

### Whole genome sequencing

Of the 82 sequenced and analyzed *Mtb* genomes, 71 provided 90% or greater coverage of the H37Rv reference genome at 10X depth (Table 2). Eight more showed intermediate (40–90%) coverage, and three showed low coverage. Remarkably, despite showing less than 10% coverage of the reference genome, Manila family Isolate 53/15RF5637 still demonstrated 200-300X coverage depth at all of the Manila specific and characteristic SNP loci. Two isolates from a putative outbreak cluster with an undefined “U” spoligotype displayed extremely low coverage. However, the three other identically fingerprinting isolates from the same putative cluster displayed >95% coverage, ensuring that the putative cluster was still able to provide data for this study.

**Table 2:** Whole genome sequencing and SNP data.

#	Accession Number	Spoligo- type	Total SNPs	Unique SNPs	Bases with 10X Coverage	Percent Coverage	Reads Passed QC	Reads Mapped	% Reads Mapped
7	09L1688	Beijing	1214	150	4366749	99.0%	8000000	7864232	98.3%
10	09L2178	Beijing	1259	5	4356699	98.8%	2157898	2122913	98.4%
16	10L8643	Beijing	1263	0	4377042	99.2%	8000000	7616623	95.2%
18	10L9505	Beijing	1199	106	4369709	99.1%	1607256	1548473	96.3%
22	11L1883	Beijing	1263	0	4297662	97.4%	912052	886821	97.2%
28	11L0426	Beijing	1254	0	4287003	97.2%	912966	897819	98.3%
29	11L0427	Beijing	1254	0	4370299	99.1%	3370522	3268479	97.0%
35	12L6290	Beijing	1263	0	4342168	98.4%	1049434	1015433	96.8%
38	12L7610	Beijing	1236	92	4338152	98.3%	1302504	1274025	97.8%
39	12L7621	Beijing	1219	126	4329373	98.1%	1001768	967945	96.6%
40	12L7991	Beijing	1236	5	4348892	98.6%	1587788	1559313	98.2%
52	15RF4481	Beijing	1224	0	4295503	97.4%	1019258	987630	96.9%
56	15RF6753	Beijing	1239	8	4269559	96.8%	868080	838900	96.6%
58	16RF0102	Beijing	1230	1	4298317	97.4%	1066562	1033295	96.9%
63	16RF2356	Beijing	1237	0	4216800	95.6%	1316206	1276147	97.0%
64	16RF4738	Beijing	1224	0	4311600	97.7%	1271118	1211113	95.3%
75	08L7125	Beijing	1231	0	4083094	92.6%	1234036	1182148	95.8%
79	10L8839	Beijing	1242	0	4284326	97.1%	1887444	1811105	96.0%
80	10L9812	Beijing	1242	0	4341917	98.4%	2928112	2806986	95.9%
81	11L3224	Beijing	1242	0	4038783	91.6%	1208578	1159426	95.9%
82	12L6284	Beijing	1264	0	4298709	97.4%	2068176	1988761	96.2%
85	13L2956	Beijing	1253	2	4339004	98.4%	3256308	3158130	97.0%
86	16RF7084	Beijing	1260	1	4340781	98.4%	2406912	2312842	96.1%
74	08L0161	Beijing	1256	1	4308269	97.7%	1918020	1858140	96.9%
77	09L2141	Beijing	1255	0	4325345	98.0%	3012910	2903739	96.4%
2	05L3477	Manila	1898	42	4382063	99.3%	1391250	1361361	97.9%
3	05L3487	Manila	1886	63	4399741	99.7%	8000000	7401374	92.5%
4	08L7789	Manila	1873	61	4381481	99.3%	1868494	1826719	97.8%
6	09L1025	Manila	1898	105	4392248	99.6%	1694468	1656631	97.8%
9	09L1898	Manila	1885	28	4375342	99.2%	1906012	1854219	97.3%
11	09L2431	Manila	1914	95	4383925	99.4%	2261522	2216486	98.0%
14	10L0115	Manila	1877	52	4374642	99.2%	2365498	2325801	98.3%
15	10L5331	Manila	1896	24	4368029	99.0%	2555006	2505891	98.1%
17	10L9503	Manila	1886	95	4396221	99.7%	8000000	7576871	94.7%
20	11L1563	Manila	1897	41	4355894	98.7%	1132140	1047235	92.5%
23	11L3658	Manila	1740	72	4386744	99.4%	3066566	3006616	98.0%
24	11L4599	Manila	1723	58	4373544	99.1%	2375178	2323296	97.8%
34	12L6289	Manila	1724	0	3096482	70.2%	506266	497032	98.2%
41	12L9260	Manila	1721	48	4352622	98.7%	2009858	1977338	98.4%
43	13L1313	Manila	1770	135	4366415	99.0%	2166864	2126405	98.1%
45	13L2435	Manila	1730	69	4346445	98.5%	1431348	1406436	98.3%
46	13L2950	Manila	1738	64	1952034	44.2%	395948	348056	87.9%
48	14RF3136	Manila	1735	49	4064968	92.1%	587692	563574	95.9%
49	14RF3267	Manila	1753	74	4210902	95.5%	719036	682961	95.0%
50	14RF5521	Manila	1755	70	2381270	54.0%	897166	279096	31.1%
51	15RF0460	Manila	1104	0	4255224	96.5%	747934	720277	96.3%
53	15RF5637	Manila	1125	37	401340	9.1%	1424204	140153	9.8%
59	16RF0108	Manila	1104	0	4274093	96.9%	876532	838126	95.6%
60	16RF1671	Manila	1121	32	3879987	88.0%	551846	531611	96.3%
61	16RF1679	Manila	1131	43	4175829	94.7%	686132	658483	96.0%
62	16RF1689	Manila	1133	64	4275638	96.9%	899476	865810	96.3%
65	16RF5423	Manila	1132	88	2048030	46.4%	859888	311073	36.2%
69	09L0735	Manila	1110	30	4356000	98.7%	1655260	1608766	97.2%
70	09L1111	Manila	1114	39	2757572	62.5%	323612	310787	96.0%
89	96128	Manila	1790	52	4370752	99.1%	4774208	4632407	97.0%
90	20034	Manila	1790	58	4360828	98.9%	2170986	2085847	96.1%
5	09L0897	EAI5	1880	190	4342065	98.4%	1479306	1428183	96.5%
33	11L5411	EAI5	1865	8	3068579	69.6%	507294	497774	98.1%
84	13L2584	no match*	1803	112	4335626	98.3%	2260038	2178054	96.4%
21	11L1565	no match**	1815	0	4361217	98.9%	1318272	1279928	97.1%
30	11L2683	no match**	1792	0	4365958	99.0%	6147928	5990291	97.4%
37	12L7424	no match**	1797	5	4352197	98.7%	2665532	2352390	88.3%
44	13L1569	no match**	1818	3	4389277	99.5%	8000000	6583222	82.3%
8	09L1897	no match	738	49	4372374	99.1%	2008844	1977757	98.5%
42	13L0560	no match	1242	574	4396157	99.7%	6411876	5888007	91.8%
71	04L0177	no match	1784	0	2273101	51.5%	550312	525881	95.6%
72	06L6898	no match	1785	0	4285043	97.1%	1880038	1799285	95.7%
73	06L7696	no match	1785	0	4229398	95.9%	1585002	1518077	95.8%
13	09L4214	H3	820	348	4361423	98.9%	2175416	2141283	98.4%
27	10L8137	H3	757	139	4097690	92.9%	615790	598982	97.3%
76	09L2140	H3	767	0	4286721	97.2%	1786962	1725623	96.6%
78	10L6616	H3	770	2	4336213	98.3%	2394498	2321661	97.0%
1	04L0176	LAM9	808	76	4376679	99.2%	2094096	2062342	98.5%
19	11L0415	T1	751	325	4358306	98.8%	1089628	1063640	97.6%
31	11L2831	T1	725	310	4385415	99.4%	8000000	7197683	90.0%
36	12L7116	T1	325	123	4393768	99.6%	1499892	1186380	79.1%
12	09L3451	T3	507	191	4382703	99.3%	2509032	2469187	98.4%
47	13RF4795	U	62	0	4294777	97.4%	891672	866742	97.2%
54	15RF5640	U	86	5	585165	13.3%	1128496	146887	13.0%
55	15RF6749	U	57	0	4224552	95.8%	796968	756386	94.9%
57	15RF6755	U	181	101	92161	2.1%	904078	55734	6.2%
83	13L2363	U	60	2	4358815	98.8%	2559816	2473275	96.6%

Isolates are identified by one or two digit University of Hawaii DNA extraction numbers and Centers for Disease Control and Prevention (CDC) genome accession numbers. Spoligotypes are named as presented in SpolDB4. Total SNPs are those identified versus the *Mtb* type strain genome H37Rv GenBank NC_000962.3 (length: 4,411,532 base pairs). Unique SNPs are alleles not found at that locus in any other isolate in this study.

*No match in SpolDB4, but strongly suspected to be Manila family based on similarity.

**Manila-like.

The numbers of SNPs displayed by each isolate are generally determined by the isolates’ families, with ancestral Manila family isolates, as expected, displaying the most SNPs against the modern H37Rv genome. The number of unique SNPs for each isolate was mostly consistent with that isolate’s clustering. Isolates from direct transmission clusters displayed zero to six unique SNPs, while isolates that shared a MIRU-VNTR fingerprint, but were not actually transmission linked (common for Beijing and Manila family isolates) displayed dozens or more.

### Identification of lineage-specific virulence factor differences

Multiple Beijing family specific virulence factor SNPs were identified in this study. In addition to identifying virulence factor differences specific to the Manila family, we also identified “lineage characteristic” virulence factor differences that were also found in a relatively uncommon non-Manila clade, but which may regardless be important for charactering the Manila family’s virulence.

#### Beijing family-specific virulence factor SNPs

Six SNPs in five previously described virulence factor genes were identified as lineage-specific non-silent virulence factor SNPs for the Beijing family (Table 3). Four additional Beijing-specific mutations in four genes possibly contributing to virulence or drug resistance, or otherwise of interest, were also identified.

**Table 3:** Beijing family-specific virulence factor SNP mutations, non-silent.

**Published Virulence Factor Mutations**											
**Nucleotide Locus**	**Reference**	**Variant**	**Rv #**	**Nucleotide #**	**Strand**	**Old Codon**	**Old AA**	**New Codon**	**New AA**	**Gene Title**	**p-value**
**213147**	C	A	Rv0182c	994	R	GAC	D	TAC	F	alternative RNA polymerase sigma factor SigG	<0.005
**213281**	C	T	Rv0182c	860	R	GGC	G	GAC	D	alternative RNA polymerase sigma factor SigG	<0.005
**1722228**	A	C	Rv1527c	6182	R	CTG	L	CGG	R	polyketide synthase Pks5	<0.005
**1877744**	A	C	Rv1661	2441	F	GAA	E	GCA	A	polyketide synthase Pks7	<0.005
**2673818**	C	G	Rv2383c	2020	R	GTG	V	CTG	L	phenyloxazoline synthase MbtB	<0.005
**3979990**	C	G	Rv3540c	670	R	GTG	V	CTG	L	lipid-transfer protein or keto acyl-CoA thiolase Ltp2	<0.005
**Possible Virulence Factor Mutations and Genes of Interest**											
**Nucleotide Locus**	**Reference**	**Variant**	**Rv #**	**Nucleotide #**	**Strand**	**Old Codon**	**Old AA**	**New Codon**	**New AA**	**Gene Title**	**p-value**
**1361285**	C	T	Rv1217c	517	R	GCT	A	AGT	S	antibiotic transporter permease	<0.005
**1643864**	T	C	Rv1458c	397	R	ACC	T	GCC	A	antibiotic transporter ATP-binding protein	<0.005
**1955941**	G	C	Rv1730c	1305	R	GAC	D	GAG	E	penicillin-binding protein	<0.005
**3127931**	T	A	Rv2820	342	R	AAA	K	AAT	N	CRISPR type III-a/mtube-associated ramp protein csm4	<0.005

List of non-silent SNPS in possible virulence factor genes or genes of interest that were exclusively found in all Beijing family isolates. None of these alleles were found in any non-Beijing-family isolates in this study. Rv numbers and gene titles are those provided by TubercuList [23]. Nucleotide loci are genomic nucleotide positions in *Mtb* H37Rv genome NC_000962.3. P-values were calculated using the Fisher Exact method. F: forward strand. R: reverse strand. AA: amino acid.

#### Manila family-specific virulence factor SNPs

Seven SNPs in six previously described virulence factor genes were identified as non-silent lineage-specific virulence factor SNPs for the Manila family (Table 4). Two of those mutations were located several bases upstream of the start codons, possibly in the promotor region or ribosome binding site. Four additional virulence factor mutations found in all Manila family isolates, but also in one epidemiological cluster with no SpoldDB4 spoligotype match and in one phylogenetically distinct EAI5 isolate, are also listed in Table 4.

**Table 4:** Family specific and characteristic virulence factor SNP mutations of the Manila family of *Mtb*.Family specific virulence factor mutations of the Manila family, non-silent.

**Nucleotide Locus**	**Reference**	**Variant**	**Rv** **#**	**Nucleotide** **#**	**Strand**	**Old Codon**	**Old AA**	**New Codon**	**New AA**	**Gene Title**	**p-value**	**#** **Non-Manila**
**200379**	C	T	Rv0170	485	F	GCC	A	GTC	V	MCE-family protein Mce1B	<0.005	-
**236512**	C	T	Rv0198c -5	-5	-	-	-	-	-	5 bases upstream Rv0198c zinc metalloprotease Zmp1, possibly in the ribosome binding site	<0.005	-
**235681**	G	A	Rv0191c	827	R	GGC	A	GTG	V	zinc metalloprotease Zmp1	<0.005	-
**495198**	A	G	Rv0410c	2117	R	TTC	F	TCC	S	serine/threonine-protein kinase PknG	<0.005	-
1038500*	T	G	Rv0931c	1415	R	CAG	Q	CCG	P	transmembrane serine/threonine-protein kinase D PknD	<0.005	-
1875295*	C	T	Pks7–9	-9	-	-	-	-	-	9 bases upstream of Rv1661 polyketide synthase Pks7, possibly in the promotor	<0.005	-
**3244126**	G	A	Rv2930	430	F	GTT	V	ATT	I	fatty-acid-AMP ligase FadD26	<0.005	-
**Family characteristic virulence factor mutations of the Manila family, non-silent**												
**Nucleotide Locus**	**Reference**	**Variant**	**Rv** **#**	**Nucleotide** **#**	**Strand**	**Old Codon**	**Old AA**	**New Codon**	**New AA**	**Gene Title**	**p-value**	**# Non-Manila**
**2745739**	G	A	Rv2445c -15	-15	-	-	-	-	-	15 bases upstream of Rv2445c nucleoside diphosphate kinase NdkA, possibly in the promotor	<0.005	2 (4)
**3244414**	A	G	Rv2930	718	F	ATG	M	GTG	V	fatty-acid-AMP ligase FadD26	<0.005	2 (4)
**3447480**	A	C	Rv3082c	947	R	CTC	L	CGC	R	virulence-regulating transcriptional regulator VirS	<0.005	2 (4)
**4290827**	C	G	Rv3823c	703	R	GGG	G	CGG	R	membrane transporter MmpL8	<0.005	2 (4)

List of family-specific and family-characteristic non-silent SNPs in virulence factor genes. All family-specific SNPs were exclusively found in all Manila family isolates. None of these Manila-specific SNPs were found in any non-Manila-family isolates in this study. Family-characteristic SNPs are non-silent SNPs in virulence factor genes that were found in all Manila family isolates, as well as a limited set of similar isolates in this study.

# Non-Manila is the number of clusters that are neither Manila family nor Manila-like that possess this allele (number of contained isolates is in parenthesis). Rv numbers and gene titles are those provided by TubercuList [23]. Nucleotide loci are genomic nucleotide positions in *Mtb* H37Rv genome NC_000962.3. P-values were calculated using the Fisher Exact method. F: forward strand. R: reverse strand. AA: amino acid. *One two-isolate Manila-like cluster did not display either of these two SNPs.

#### Manila family-specific possible virulence factor or gene-of-interest SNPs

Five Manila-specific SNPs in genes possibly contributing to virulence or drug resistance, or otherwise of interest, were identified (Table 5). Six additional such mutations found in all Manila family isolates, but also in one epidemiological cluster with no SpoldDB4 spoligotype match and in one phylogenetically distinct EAI5 isolate, are also listed in Table 5.

**Table 5:** Manila family specific and characteristic SNP mutations in possible virulence factor genes and genes of interest.

**Family specific possible virulence factor mutations and genes of interest of the Manila family, non-silent**												
**Nucleotide Locus**	**Reference**	**Variant**	**Rv** **#**	**Nucleotide** **#**	**Strand**	**Old Codon**	**Old AA**	**New Codon**	**New AA**	**Gene Title**	**p-value**	**# Non-Manila**
**27199**	G	A	Rv0022c	244	R	CAG	Q	TAG	STOP	transcriptional regulator WhiB-like WhiB5	<0.005	-
**2239160**	G	C	Rv1996	157	F	GGG	G	CGG	R	universal stress protein	<0.005	-
**2654696**	G	A	Rv2374c	398	R	TCG	S	TTG	L	heat shock protein transcriptional repressor HrcA	<0.005	-
**3129675**	C	T	Rv2823c	2099	R	CGC	R	CAC	H	CRISPR-associated protein cas10/csm1, subtype III-a/mtube	<0.005	-
**27473**	G	T	WhiB5–31	-31	-	-	-	-	-	31 bp upstream of transcriptional regulator WhiB-like WhiB5, possibly in the promotor	<0.005	-
**Family characteristic possible virulence factor mutations and genes of interest related to the Manila family, non-silent**												
**Nucleotide Locus**	**Reference**	**Variant**	**Rv** **#**	**Nucleotide** **#**	**Strand**	**Old Codon**	**Old AA**	**New Codon**	**New AA**	**Gene Title**	**p-value**	**# Non-Manila**
**412280**	T	G	Rv0342	1443	F	CAT	H	CAG	Q	isoniazid inducible protein IniA	<0.005	2 (4)
**1097023**	G	A	Rv0981	202	F	GGC	G	AGC	S	mycobacterial persistence regulator MRPA	<0.005	2 (4)
**2239055**	C	T	Rv1996	52	F	CCC	P	TCC	S	universal stress protein	<0.005	2 (4)
**2574598**	C	T	Rv2303c	422	R	AGC	S	AAC	N	antibiotic-resistance protein	<0.005	2 (4)
**2726051**	G	A	Rv2427ac	37	-	-	-	-	-	pseudogene OxyR protein	<0.005	2 (4)
**27469**	A	G	WhiB5–27	-27	-	-	-	-	-	27 bp upstream of transcriptional regulator WhiB-like WhiB5, possibly in the promotor	<0.005	2 (4)

List of family-specific and family-characteristic non-silent SNPs in possible virulence factor genes or genes of interest. All family-specific SNPs were exclusively found in all Manila family isolates. None of these Manila-specific SNPs were found in any non-Manila-family isolates in this study. Family-characteristic SNPs are non-silent SNPs in virulence factor genes that were found in all Manila family isolates, as well as a limited set of similar isolates in this study.

# Non-Manila is the number of clusters that are neither Manila family nor Manila-like that possess this allele (number of contained isolates is in parenthesis). Rv numbers and gene titles are those listed in TubercuList [23]. Nucleotide loci are genomic nucleotide positions in *Mtb* H37Rv genome NC_000962.3. P-values were calculated using the Fisher Exact method. F: forward strand. R: reverse strand. AA: amino acid

### Relationship of Manila-like isolates to the Manila family

**Fig 1:**
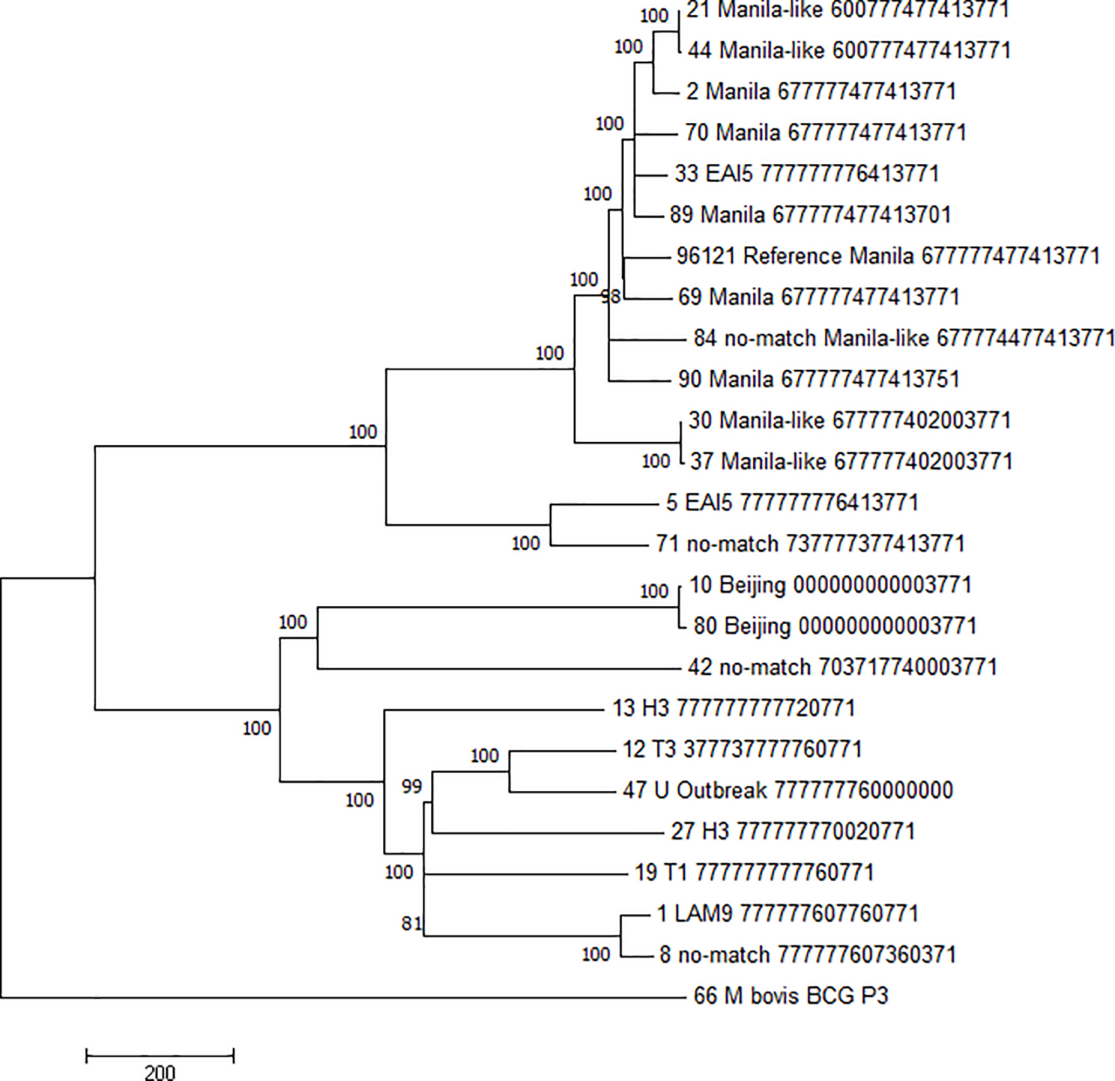
Maximum parsimony phylogeny of Manila-like isolates. Evolutionary history inferred by MEGA7 using the maximum parsimony method and rooted by *Mycobacterium bovis* BCG P3. Branch support was determined by 1000 bootstrap repetitions (bootstrap values shown above each node). A model averaged phylogeny further supported this result (data not shown).

Phylogenetic analysis based on all genomic SNPs among selected isolates was conducted to further investigate the relationships of “Manila-like” and other strains to the Manila family (Fig 1). This phylogeny showed that the Manila family isolates that were selected due to their uncommon (but still Manila family) spoligotyping patterns (isolates 89 and 90) grouped within Manila family isolates that displayed the common Manila pattern. Likewise, the isolates selected based on previous deletion analysis to maximize the Manila family diversity in this study (isolates 69 and 70) grouped with other Manila family isolates as expected.

Other isolates displayed diverse relationships with the Manila family. One cluster of “Manila-like” isolates whose spoligotypes had no match in SpolDB4 (isolates 21 and 44) were shown to group inside the Manila family branch. However, one no-match Manila-like cluster (isolates 30 and 37), which was also observed to exhibit pigmentation when grown on Löwenstein–Jensen medium and differed from the Manila family at only two virulence factor SNP loci, occupies a branch of the phylogeny immediately divergent to the Manila family. Interestingly, one EAI5 isolate (5) and a three-isolate no SpolDB4 match epidemiological cluster (71–73) occupy a distinct branch between lineage 1 (Manila family and other EAI lineages) and the modern lineages that include lineages 2 (including the Beijing family) and 4 (including the LAM lineages, H lineages, and others). This was also an expected result, as those isolates matched the Manila-characteristic virulence factor SNP alleles and deviated from them in roughly equal number (Table 6). Other isolates with no SpolDB4 match (isolates 8 and 42, selected for diversity due to appearing to belong to no obvious lineage) grouped with lineage 4 isolates.

**Table 6:** Virulence factor SNPs specific to Manila and Manila-like isolates.

Spoligotype Name	*Manila Reference*	Uncommon Manila Spoligotype	EAI5	Uncommon Manila Spoligotype	Manila-like*	Manila-like**	Manila-like** ^†^	No-SpolDB4 Match^‡^	EAI5	No SpolDB4 Match	Beijing (Outgroup)	No SpolDB4 Match	*Mtb Type Strain*
**Isolate #**	***96121***	**89**	**33**	**90**	**21**	**84**	**37**	**71**	**5**	**8**	**10**	**42**	***H37Rv***
**Spacers Lost**	-	3	1	1	6	2	7	2	1	8	26	13	3
**Spacer Gained**	-	-	3	-	-	-	-	3	3	6	0	1	5
**SNPs vs. 96121**	-	**239**	**239**	**278**	**281**	**299**	**437**	**872**	**1020**	**1920**	**1964**	**1970**	**NA**
27,469	G	G	G	G	G	G	G	G	G	A	A	A	A
412,280	G	G	G	G	G	G	G	G	G	T	T	T	T
1,097,023	A	A	A	A	A	A	A	A	A	G	G	G	G
2,239,055	T	T	T	T	T	T	T	T	T	C	C	C	C
2,574,598	T	T	T	T	T	T	T	T	T	C	C	C	C
2,726,051	A	A	A	A	A	A	A	A	A	G	G	G	G
2,745,739	A	A	A	A	A	A	A	A	A	G	G	G	G
3,244,414	G	G	G	G	G	G	G	G	G	A	A	A	A
3,447,480	C	C	C	C	C	C	C	C	C	A	A	A	A
4,290,827	G	G	G	G	G	G	G	G	G	C	C	C	C
27,199	A	A	A	A	A	A	A	G	G	G	G	G	G
27,473	T	T	T	T	T	T	T	G	G	G	G	G	G
200,379	T	T	T	T	T	T	T	C	C	C	C	C	C
235,681	A	A	A	A	A	A	A	G	G	G	G	G	G
236,512	T	T	T	T	T	T	T	C	C	C	C	C	C
495,198	G	G	G	G	G	G	G	A	A	A	A	A	A
2,239,160	C	C	C	C	C	C	C	G	G	G	G	G	G
2,654,696	A	A	A	A	A	A	A	G	G	G	G	G	G
2,673,701	T	T	T	T	T	T	T	C	C	C	C	C	C
3,129,675	T	T	T	T	T	T	T	C	C	C	C	C	C
3,244,126	A	A	A	A	A	A	A	G	G	G	G	G	G
1,038,500	G	G	G	G	G	G	T	T	T	T	T	T	T
1,875,295	T	T	T	T	T	T	C	C	C	C	C	C	C

Comparison of SNP alleles between Manila family, Manila-like, unknown, and outgroup isolates. Shaded alleles match those displayed by *Mtb* type strain H37Rv, while non-shaded alleles match representative Manila family isolate 96121. Loci displayed in the lower two groups are found in Tables 4 and 5 as family-specific SNPs. Loci in the upper group are found in Tables 4 and 5 as family-characteristic SNPs; although not exclusive to the Manila family, these SNPs shared only with isolates from outside of existing major spoligotyping lineages may be important for defining virulence of the Manila family. All Manila family diversity selection isolates present the same alleles, as do two Manila-like clusters and one EAI5 isolate. One Manila-like cluster differed from the Manila family at two of these selected loci. One other EAI5 isolate and one cluster with no SpolDB4 match diverged from the Manila family at thirteen selected loci. These differences suggest possible differences in virulence for this small clade.

Nucleotide loci are genomic nucleotide positions in *Mtb* H37Rv genome NC_000962.3.

Spacers gained or lost are relative to the Manila family reference spoligotype 677777477413771.

* No match in SpolDB4, but Manila-like; representative of a two-isolate epidemiological cluster with both isolates sharing the same alleles at these loci.

**No match in SpolDB4, but almost certainly Manila family; representative of a two-isolate epidemiological cluster with both isolates sharing the same alleles at these loci.

† Representative of a two-isolate epidemiological cluster with both isolates sharing the same alleles at these loci.

‡ Representative of a three-isolate epidemiological cluster with all isolates sharing the same alleles at these loci.

Table 6 further illustrates the distribution of loci that were designated as either Manila-specific (found only in Manila family isolates) or Manila-characteristic (found in all Manila family isolates as well as certain related groups). Notably, two SNPs (1,038,500 and 1,875,295) that were designated as Manila-specific were not shared by one two-isolate Manila-like cluster (isolates 30 and 37), though the cluster matched the Manila family at the other virulence loci. Additionally, since octal coding might not intuitively convey how many spacers are gained or lost with a change from one digit to another, Table 7 presents the numbers of spacers gained or lost by each isolate relative to Manila family reference genome 96121. This comparison of SNPs versus 96121 and spacers gained or lost clearly illustrates that the number of spacers lost by an isolate relative to 96121 fails to predict an isolate or lineage’s closeness to the Manila family.

**Table 7:** Clustering and spoligotype details of selected Manila and Manila-like isolates.

96121 vs isolate #	SNPs vs. 96121	Isolate Cluster	Spoligotyping Pattern (96121: 677777477413771)	Spacers Lost	Spacers Gained
**70**	193	Manila Diversity Selection (Deletions)	677777477413771	-	-
**69**	210	Manila Diversity Selection (Deletions)	677777477413771	-	-
**89**	239	Manila Diversity Selection	6777774774137**0**1	3	-
**33**	239	EAI5	**7**77777**7**7**6**413771	1	3
**2**	278	Manila Cluster A	677777477413771	-	-
**90**	278	Manila Diversity Selection	6777774774137**5**1	1	-
**21**	281	Manila-like Cluster 1	6**00**777477413771	6	-
**44**	293	Manila-like Cluster1	6**00**777477413771	6	-
**84**	299	Manila-like Cluster B	67777**4**477413771	2	-
**37**	437	Manila-like Cluster 2 (Pigmented)	6777774**0200**3771	7	-
**30**	438	Manila-like Cluster 2 (Pigmented)	6777774**0200**3771	7	-
**71**	872	02–06 No SpolDB4 Match	**73**7777**3**77413771	2	3
**72**	873	02–06 No SpolDB4 Match	**73**7777**3**77413771	2	3
**73**	873	02–06 No SpolDB4 Match	**73**7777**3**77413771	2	3
**5**	1020	EAI5	**7**77777**7**7**6**413771	1	3
**8**	1920	Diversity Selection	**7**77777**60**7**360**371	8	6
**80**	1925	Beijing Family (Outgroup)	**00000000000**3771	26	0
**10**	1964	Beijing Family (Outgroup)	**00000000000**3771	26	0
**42**	1970	Diversity Selection	**703**7**1**774**000**3771	13	1

Details of the clusters represented by isolates in Fig 1. Spoligotyping octal elements that differ from the standard Manila family spoligotyping pattern 677777477413771 are marked in bold and underlined, with the numbers of CRISPR spacers gained or lost (versus the standard Manila family pattern) also presented. Isolates are ordered by the total number of genomic SNPs against *de novo* sequenced representative Manila family isolate 96121. Note that the number of spacers lost relative to the Manila family reference fails to indicate the closeness of the isolate’s relationship to 96121, as determined by the number of SNPs between them.

## Discussion

### Identification of lineage-specific virulence factor differences

Lineage-specific SNPs in virulence factor genes were identified for both the Beijing and Manila families. These were defined as non-silent mutations that were shared by all isolates in that family, but could not be found outside of that family in this study.

#### Lineage-specific Beijing family virulence factor SNPs

This study identified several virulence factor mutations specific to the Beijing family. Two mutations were found in alternative RNA polymerase sigma factor gene *sigG*, which has been shown to be upregulated during macrophage infection as well as by DNA damage, despite not regulating DNA repair genes. Accordingly, its deletion has been shown to impair survival in a macrophage infection model [24, 25]. Mutations were also found in polyketide synthase genes *pks5* and *pks7*. The contribution of these genes to *Mtb* virulence has been investigated by multiple studies. For example, disruption of Pks5 showed no difference in cell envelope lipid composition, but resulted in severe growth defects in a mouse infection model. In contrast, disruption of Pks7 produced a strain that was deficient in the production of phthiocerol dimycocerosates (PDIMs), which has been known to attenuate growth during *in vivo* infection, and in this case attenuated growth in mice infected by respiratory inoculation [18, 26, 27]. In addition, W-Beijing family strains have been shown to produce a polyketide synthase-derived phenolic glycolipid and demonstrated hypervirulence in a murine model, associated with a reduction in production of pro-inflammatory cytokines TNF-α, IL-6, and IL-12 [28]. One mutation was found in phenyloxazoline synthase MbtB, which is involved in iron acquisition, an important virulence factor for growth inside a macrophage, where iron levels can be 1/1000^th^ those outside the leukocyte [18]. The final Beijing family virulence factor found to display a lineage-specific mutation was lipid-transfer protein or keto acyl-CoA thiolase Ltp2, which may be involved in cholesterol transport [18].

The Beijing family’s lineage-specific mutations in other genes of interest included one in antibiotic transporter permease Rv1217c, which has been shown to actively transport the ionophore antibiotic tetronasin across the cell membrane, and one in antibiotic transporter ATP-binding protein Rv1458c, which is similar to other described antibiotic transporters [29]. A mutation was also found in a penicillin-binding protein (Rv1730c). Penicillin is widely regarded as ineffective against *Mtb*, but the rise of extensively drug-resistant TB has renewed interest in the use of beta-lactam drugs against it [30]. Additionally, studies combining beta lactam drugs with beta lactamase inhibitors have shown promise [31]. Finally, CRISPR type III-a/mtube-associated ramp protein Csm4 showed one mutation. Although the exact role of Csm4 is still being deduced, CRISPR-related changes in the Beijing family are of interest due to its lack of many of the CRISPR spacers from the set of 43 utilized by spoligotyping that are present in many other lineages.

The Beijing family has been shown to be split into ancient (atypical) and modern (typical) sublineages on the basis of the presence or absence of one to two IS*6110* insertion sequences in the NTF region, with the modern sublineage being shown to display increased virulence relative to the ancestral sublineage [32]. Depending on their sublineage, Beijing family isolates display mutations in two DNA repair genes (*mutT4* and *mutT2*) as well as *ogg*, which are thought to result in an increased mutation rate, allowing for quicker adaptation to new host pressures or environments [32, 33]. The genetic difference between ancient and modern Beijing strains has been further explored, and while mutations have been identified, their phenotypic results still require investigation [34]. Our present study, however, did not seek to include isolates of the less clinically important ancient (atypical) clade. A previous study identified a set of 81 SNPs that characterized all modern Beijing family strains [35]. That study identified virulence factor SNPs in the *mce* and *vapBC* gene families, but our study indicated that those SNPs are not specific to the Beijing family (data not shown). Another study that sought to characterize the modern/typical Beijing lineage identified 51 SNPs that defined the lineage and were not found in a search of 29 non-Beijing strains [36]. Notably, their set of SNPs found regulatory network mutations to be over-represented. As our study only searched for mutations in established virulence factor genes, mutations in regulatory genes that might be responsible for major differences in virulence would not have been captured by this study unless those regulatory genes had already been characterized as virulence factors.

#### Lineage-specific Manila family virulence factor SNPs

The Manila family exhibited a similar number of lineage-specific virulence factor mutations as the Beijing family. However, we also identified a group of isolates (EAI5 isolate 5 and a cluster with no SpolDB4 match composed of isolates 71–73) that showed virulence factor SNPs matching the Manila family at some loci, but matching an outgroup at others. Those SNPs that were found only in the Manila family and in this small, ancient clade were presented as Manila family characteristic SNPs rather than Manila family specific SNPs. While not 100% specific to the family, these mutations may still be important for understanding the virulence or transmission characteristics of the Manila family.

Among the notable virulence factor genes hosting the Manila family’s lineage specific SNPs, one mutation was found in *mce1B*. One study showed that when the *mce1* operon was knocked out, the resulting infection was unable to enter a state of persistent infection in mouse lungs, instead exhibiting hypervirulence and killing the mice more rapidly than wild-type *Mtb*. From *ex vivo* infection of murine macrophages, this was proposed to have resulted from the mutant failing to stimulate a Th-1 mediated immune response that would have induced protective granuloma formation [37]. One Manila family-specific mutation was found five bases upstream of the zinc metalloprotease *zmp1* start codon, which might place it within the ribosome binding site (RBS). Mycobacterial ribosome binding sites are typically G and A-rich regions of six to eight base pairs in length, located four to seven base pairs upstream of the start codon [38]. Zmp1 is further of interest as it contains a second Manila family specific mutation, and *zmp1*’s deletion has been shown to result in hypervirulence in a C57BL/6 mouse model [39]. One mutation was found in the gene encoding PknG, which is secreted into the macrophage cytosol upon mycobacterial infection, where it is required for inhibition of phago-lysosomal fusion and mediates persistence under stressful conditions [40, 41]. Another mutation was found in *pknD*, which is required for invasion of brain endothelia, but not lung epithelia or macrophages [42]. While the Beijing family displayed a family-specific mutation in *pks7*, the Manila family displayed a family-specific mutation nine bases upstream of the gene, possibly affecting the promotor or ribosome binding site. Finally, the Manila family exhibits a mutation in *fadD26*, whose disruption results in impaired synthesis of phthiocerol dimycocerosates and attenuation in a mouse model [43]. Interestingly, one potential *Mtb* vaccine that has now completed a phase I clinical trial, MTBVAC, was produced from a double deletion mutant that removed *phoP* and *fadD26* [44].

Manila family characteristic mutations were found in several additional genes. Nucleoside diphosphate kinase NdkA inactivates GTPase Rac1 in macrophages (which reduces production of reactive oxygen species), and knock down of *ndkA* resulted in increased susceptibility to intracellular killing and reduced persistence in the lungs of infected mice [45]. Another mutation was also observed for *fadD26* (in addition to the family-specific mutation), suggesting that FadD26 may be important in directing the virulence of the Manila family. VirS is a transcriptional regulator controlling the *mymA* operon, which is induced by infection in macrophages, and may be involved in remodeling *Mtb*’s cellular envelope under acidic, intracellular conditions [46]. Finally, a mutation was found in *mmpL8*, disruption of which resulted in attenuation presenting as significantly increased time to death in a mouse aerosol infection model, corresponding to less efficient suppression of cytokines directing a Th1-type immune response [47].

SNPs in genes of interest were likewise split between lineage-specific and lineage-characteristic for the Manila family. The most notable lineage-specific gene was transcriptional regulator *whiB5*, which influences the expression of 58 genes, and which our study revealed is truncated due to a premature stop codon in the Manila family [48]. Both chronic and progressive infections have been found to be attenuated in a mouse model when *whiB5* was deleted. The mutant was also shown to be unable to resume growth after reactivation from chronic infection. However, as previously noted, human infections with Manila family strains do not appear to display any deficiency in reactivation [49]. Furthermore, two mutations were observed upstream of *whiB5*’s start codon (-27 and -31). These mutations may be located in the -35 promoter sequence, but that is difficult to determine as mycobacteria have little homology in their -35 promoter sequences [38]. Although studies have shown that the -35 region is not essential for promoter function in mycobacteria, manipulation of the region can vary the wild-type promotor activity from as little as 6% to as much as 200% of its original activity [38]. Regardless, it is interesting that while the *whiB5*–31 mutation was observed only in Manila family isolates, a *whiB5*–27 mutation was observed in the Manila family, both EAI5 clades, and the ancient three-isolate epidemiological cluster with no SpolDB4 match. Another gene of interest is the *oxyR* pseudogene. *oxyR* nucleotide 37 T has been used as a marker for the Manila family and EAI5 lineage [50]. However, this study revealed that *oxyR* 37 T is not fully exclusive to the Manila family, as the newly identified ancient clade with an EIA5 isolate and a no SpolDB4 match cluster also exhibited the mutation. Finally, a Manila family characteristic mutation was observed in mycobacterial persistence regulator MRPA, which of *Mtb*’s ~214 transcriptional regulators, was one of seven shown to be upregulated during nonreplicating persistent stage stage 2 (NRP2) [51]. MRPA had been previously shown to be required for entrance into and maintenance of persistent infection [52].

### Relationship of Manila-like isolates to the Manila family

Full genome sequencing provides insight here into the connection of isolates with putative “Manila-like” spoligotypes to the Manila family. A phylogenetic tree determined from those full genome SNP sets has further established their relationships (Fig 1).

In general, the number of spoligotyping/CRISPR spacers lost by an isolate relative to the Manila family did not serve as a predictor of the number of total SNP differences between that isolate and reference Manila family isolate 96121 (Table 7). Multiple examples demonstrating this are presented below. Manila family isolate 89, with its three missing spacers relative to the Manila family’s prototypical pattern, displayed the same number of SNPs from 96121 as EAI5 isolate 33 (which lost one spacer and gained three). Furthermore, both of those isolates had fewer SNPs versus 96121 than isolate 2 (chosen to represent a large Manila family epidemiological cluster). The epidemiological cluster represented by isolates 21 and 41 was missing six spacers, but did not display many more SNPs versus 96121. However, other than for EAI5, gain of spacers relative to the Manila family appeared to be a better indication of distance from the Manila family than loss of spacers. EAI5 isolate 5 and a three-isolate no SpolDB4 match epidemiological cluster (isolates 71–73) occupy a branch together, with both gaining the same three spacers, but the one spacer lost by isolate 5 and the two spacers lost by the 71–73 cluster do not overlap. Other isolates whose spoligotypes had no match in SpolDB4 differed from Manila by the largest numbers of SNPs, similar to the Beijing family outgroup isolates. Of those no-match isolates, isolate 8 was selected due to having a large number of spacers gained versus 96121, and it clustered closest to LAM9 isolate 1, while isolate 42, selected to represent a large number of spacers lost, clustered closest to the Beijing family isolates (despite the apparent difference in spoligotypes).

The numbers of SNPs between individual isolates and our Manila family reference genome further clarify the relationships of isolates displaying various spoligotyping patterns to the Manila family. Isolates 69 and 70, selected from a prior study (which used deletion-based analysis) in order to maximize our present study’s diversity within the Manila family, expectedly clustered within the other Manila family isolates, and displayed the fewest SNPs versus 96121 (210 and 193 SNPs, respectively). Manila family isolate 89, selected due to losing three of the Manila family’s characteristic spoligotyping spacers, likewise clustered with the rest of the Manila family, while Manila family isolate 90 (which lost only a single spacer) was more distinct, and clustered closer to isolate 84 (which lost two spacers and had no SpolDB4 match). Another un-matched, but seemingly Manila-like spoligotype displayed by epidemiologically clustered isolates 21 and 44 (missing seven spacers) clustered within the Manila family. An additional un-matched, but seemingly Manila-like, epidemiological cluster of isolates 30 and 37 (missing seven spacers) clustered furthest from the Manila family, yet still on a distinct branch from the Manila family’s known outgroups. Despite this, it was the only “Manila-like” group not to match the Manila family in all of the virulence factor mutations that characterized the family in this study, differing in a SNP at transmembrane serine/threonine-protein kinase D *pknD* and a SNP 9 bases upstream of Rv1661 polyketide synthase *pks7*, possibly in the promotor. Furthermore, isolates from this cluster were also observed to exhibit pigmentation when grown on Löwenstein–Jensen medium.

The EAI5 spoligotype revealed interesting results. We have previously identified in other EAI5 isolates that this spoligotype covers both Principle Genetic Groups 1 and 2 [53]. Thus, this EAI5 spoligotype spans evolutionarily distinct clades, possibly as the result of convergent evolution of CRISPR regions due to phage pressure. Both EAI5 isolates in this study were found to be ancient, despite both phylogenetics and virulence SNPs showing them to be quite distinct. While EAI5 isolate 33 clustered with the Manila family, EAI5 isolate 5 clustered with a three-isolate epidemiological cluster with no SpolDB4 match (isolates 71–73). This sub-clade of ancient isolates was roughly split between matching the Manila family and matching outgroup H37Rv at the Manila family’s lineage specific and characteristic SNP loci (10 to 13). Some notable virulence factor differences for those isolates include losing the Manila family’s truncated transcriptional regulator WhiB5 and one of its two upstream mutations, SNPs in MCE-family protein Mce1B, zinc metalloprotease Zmp1 and its upstream mutation, and one of the two fatty-acid-AMP ligase FadD26 mutations, among others (Table 6). These differences suggest that this ancient sub-clade displays virulence characteristics different from the Manila family.

## Conclusions

This study identified distinct sets of non-silent virulence factor SNPs that were specific to the Beijing and Manila families of *Mtb*. Further study of these mutations could lead to greater understanding of differences in virulence in these two families. Future work investigating the effects of these SNPs in animal or macrophage infection models, which is beyond the scope of this present study, will be required to fully determine their phenotypes. However, future studies should not be limited to investigating only single SNPs. *Mtb* lacks singular genomic features that account for host preference, and epistatic interactions and coordination of transcriptional regulation must be considered together to account for *Mtb*’s transmission and virulence [5]. Thus, investigating only single SNPs from a specific family alone (in the absence of the family’s other mutations) may be insufficient to produce a phenotype with altered virulence. Additionally, this study utilized only a small (82-isolate) set of Hawaii-focused *Mtb* isolates. Future work will further establish the lineage-specificity of these mutations by significantly expanding the number of genomes investigated. Although repositories such as the National Center for Biotechnology Information (NCBI) GenBank already hold a large number of *Mtb* genomes, the lack of paired spoligotyping data attached to those genomes limits their use in this study. A tool for *in silico* spoligotyping of *Mtb* genomes, SpolPred, enables identification of spoligotypes from FASTQ sequencing read files, allowing data from repositories such as the NCBI Sequence Read Archive (SRA) to be investigated [54]. However, while Beijing family sequencing data is abundant, Manila family sequencing data currently remains uncommon.
